# Supplementary material for: Multi-Level Analysis of Adipose Tissue Reveals the Relevance of Perivascular Subpopulations and an Increased Endothelial Permeability in Early-Stage Lipedema
Source: Biomedicines. 2022 May 18;10(5):1163. doi: 10.3390/biomedicines10051163 (PMC9138324; doi:10.3390/biomedicines10051163)
Supplement: Supplementary file 1 [file biomedicines-10-01163-s001.zip › Supplementary Figure S13.pdf]

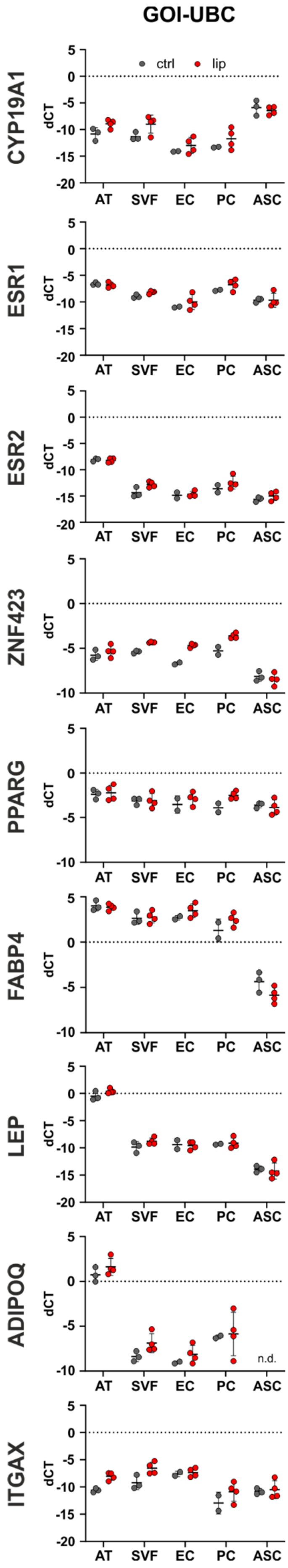

GOI-UBC
ctrl
lip
CYP19A1
ESR1
ESR2
ZNF423
PPARG
FABP4
LEP
ADIPOQ
ITGAX
dCT
AT
SVF
EC
PC
ASC
n.d.

Supplementary Figure S13. Gene expression analysis of factors involved in local estrogen metabolism, adipogenesis and inflammation. Subcutaneous adipose tissue (AT) of the thigh region collected from healthy individuals (ctrl: n=3) and lipedema patients (lip-thigh: n=4), as well as abdominal AT of lipedema patients (lip-abdomen: n=3) served to isolate the stromal vascular fraction (SVF), which was in turn sorted for CD31+ endothelial cells (EC) and CD45- CD31- CD146+ pericytes (PC). SVF was cultivated and passaged two times to collect adipose-derived stromal/stem cells (ASC P2). RNA was isolated and quantitative real-time RT-PCR for gene expression analysis was performed. Expression levels of gene of interest (GOI) compared to housekeeping gene, Ubiquitine C (UBC), are displayed as dot plots of delta cycle threshold (dCT) values, mean ± SD. Abbreviations: CYP19A1: Cytochrome P450 family 19 subfamily A member 1, Aromatase; ESR1: Estrogen receptor 1; ESR2: Estrogen receptor 2; ZNF423: Zinc finger protein 423; PPARG: Peroxisome proliferator-activated receptor gamma; FABP4: Fatty acid binding protein 4; LEP: Leptin; ADIPOQ: Adiponectin; ITGAX: Integrin subunit alpha X, CD11C, n.d.: Not detected.
